# Expressions of Carbohydrate Response Element Binding Protein and Glucose Transporters in Liver Cancer and Clinical Significance

**DOI:** 10.1007/s12253-019-00708-y

**Published:** 2019-08-12

**Authors:** Yu Lei, Qiaoling Hu, Jiang Gu

**Affiliations:** 1 Department of Pathology and Pathophysiology, Provincial Key Laboratory of Infectious Diseases and Immunopathology, Collaborative and Creative Center, Shantou University Medical College, Shantou, 515041 Guangdong China; 2 Department of Pediatrics, University of Groningen, University Medical Center Groningen, 9713 GZ Groningen, The Netherlands; 3 Jinxin Research Institute for Reproductive Medicine and Genetics, Chengdu Jinjiang Hospital for Maternal and Child Health Care, 66 Jingxiu Road, Chengdu, 610066 China; 4 Department of Pathology, Beijing University Health Science Center, Beijing, 100083 China

**Keywords:** ChREBP, Glucose transporters, Hepatocellular carcinoma, Diagnostic marker, Glycolysis

## Abstract

Carbohydrate response element binding protein (ChREBP) is a glucose-sensing transcription factor that mediates the induction of glycolytic and lipogenic genes in response to glucose. We investigated the expression patterns of ChREBP and glucose transporters (GLUTs) in human hepatocellular carcinoma (HCC) and their association with HCC progression. ChREBP, GLUT2 and GLUT1 immunohistochemistry were performed on liver tissue array containing normal liver tissue, HCC adjacent tissue and cancer tissue of different HCC stages. The effect of HCC malignancy on protein expression was analyzed with one-way ANOVA. The correlations between protein expressions were analyzed with Pearson Correlation test. We found that ChREBP protein expression tended to be positively correlated to liver malignancy. GLUT2 protein expression was significantly reduced in human HCC as compared to normal liver tissue and its expression in HCC was inversely associated to malignancy (*p* < 0.001). In contrast, GLUT1 was significantly increased in cancer cells and its expression was positively correlated to malignancy (*p* < 0.001). Furthermore, GLUT1 expression was positively associated to ChREBP expression (r = 0.481, *p* < 0.0001, *n* = 70) but negatively correlated to GLUT2 expression (r = −0.320, *p* = 0.007, n = 70). Notably, ChREBP-expressing hepatocytes did not express GLUT2 but GLUT1. This is the first report unveiling expressions of ChREBP and GLUT2/GLUT1 and their relations in HCC. The expression patterns are related to malignancy and this information would facilitate evaluation of clinical behavior and treatment of HCC.

**Electronic supplementary material:**

The online version of this article (10.1007/s12253-019-00708-y) contains supplementary material, which is available to authorized users.

## Introduction

Hepatocellular carcinoma (HCC) is the fifth most common cancer in men and the seventh in women. Many HCC patients have progressed to advanced stages at time of diagnosis, resulting in poor prognoses and high mortality. Despite various treatment options, survival of HCC is poor due to late diagnosis and resistance to chemotherapy. The current gold standard and most commonly used diagnostic marker for HCC is plasmic alpha-fetoprotein (AFP) along with ultrasound every 6 to 12 months, but this is far from satisfactory. Only serum AFP levels of more than 400 ng/mL are considered truly diagnostic, but such high values are observed only in a small percentage of patients with HCC at advanced stages [1]. Although many molecules have been considered to associate with diagnosis and prognosis, their presumed merits are controversial [2, 3]. Accordingly, new diagnostic and prognostic factors are needed to improve HCC diagnosis and treatment.

Evidence is accumulating that perturbed cellular metabolism predisposes human to tumor development. Metabolic diseases such as obesity and diabetes are associated with increased risk to develop various cancers [4–6]. In addition, many human tumors display a high rate of aerobic glycolysis, de novo fatty acid synthesis and nucleotide biosynthesis [7, 8]. It has been proposed that increased glucose metabolism promotes lipogenesis and nucleotide biosynthesis, and enhances tumor cell growth and proliferation by providing essential synthetic and bioenergetic molecules [9, 10].

Cancer cells acquire energy from glucose to satisfy their high metabolic demands. Glucose and related hexoses are transported into cells via glucose transporter (GLUT) family proteins (Solute carrier, SLC2A family) [11]. To date, 14 GLUT family members have been identified, each of them with different affinities for glucose and their expression patterns are tissue dependent [12, 13]. Many studies have demonstrated that the expression of glucose transporters, especially GLUT1, is increased in a variety of tumors, including pancreatic, breast, esophageal, and brain cancers [14, 15]. GLUT1 overexpression is associated with tumor progression and poor overall survival in various malignant tumors [16, 17]. GLUT2 is expressed in liver, pancreatic islet cells and retina and it is the most abundant glucose transporter in liver [18]. GLUT2 mRNA expression was shown to be increased in gastric tumors [19] but decreased in insulinomas when compared to normal tissues [20]. However, data on these glucose transporter expression in different stages of HCC have been lacking.

One of the master regulators of intracellular glucose metabolism is carbohydrate responsive element binding protein (ChREBP), a basic helix–loop–helix leucine zipper (bHLH-LZ) transcription factor expressed in liver, white and brown adipose tissues, intestine, muscle, and pancreatic β-cells [21, 22]. Elevated insulin, in response to increased glucose levels, promotes ChREBP dephosphorylation and its translocation from the cytoplasm to the nucleus where, in association with its binding partner MLX (Max-like interacting protein), it binds to carbohydrate response elements, present in the promoters of target genes. Glucose metabolites F6P and acetyl-CoA can increase ChREBP activity via O-linked glycosylation and acetylation, respectively [23–25]. The ChREBP/Mlx heterodimer controls glucose and lipid metabolism through regulating glycolytic (*Pklr, Fk, Glut2, Glut4*), gluconeogenic (*G6pc*), and lipogenic (*Fasn, Acc1, Scd1, Elovl6*) gene expressions [22, 26, 27], suggesting that ChREBP may have an important role in the pathogenesis of metabolic diseases and cancer. So far, most of the work on ChREBP has focused on its function as a hepatic transcription factor, its activation by glucose metabolites and its role in the regulation of lipogenesis. Little is known about the role of ChREBP in cancer cells. Genomic analysis of ChREBP target gene expression in human hepatocellular carcinoma cell line HEPG2 by ChIP-sequencing showed that ChREBP regulates genes associated with a tumor metabolic phenotype and malignant progression, such as tumor differentiation and motility [28]. Suppression of ChREBP in hepatocellular carcinoma and colorectal cancer cells led to reduced lipogenesis and nucleotide synthesis and decreased proliferative and tumorigenic potential in mice [29]. In breast cancer, the level of ChREBP protein expression is positively correlated with tumor progression [30]. However, hepatic ChREBP expression has not been examined in human HCC and the relationship between ChREBP expression and the degree of liver tumor malignancy has yet to be investigated.

In this study, we analyzed GLUT family members GLUT2 and GLUT1, and ChREBP protein expression levels in human liver tissue array composed of normal, HCC and adjacent liver tissue. We found that the expression of ChREBP showed a tendency to increase with liver malignancy, but unexpectedly, that GLUT2 protein expression was decreased in cancer cells compared to normal hepatocytes and its expression was negatively associated with advanced stages of HCC. GLUT1 was increased in cancer tissues and its expression was significantly correlated with clinical stage. Moreover, ChREBP and GLUT1 expressions were positively correlated to each other but GLUT1 and GLUT2 expressions were negatively correlated. Therefore, a combined evaluation of ChREBP, GLUT1 and GLUT2 protein expression profile may provide a new diagnostic and prognostic marker for HCC, that might be useful in improving patient treatment and survival.

## Materials and Methods

Human hepatic carcinoma and normal tissue microarray was purchased from AURAGENE (TC0145, Changsha, China). The tissue array contains 40 cases of hepatocellular carcinoma, 17 cases of normal tissue and 13 cases of adjacent normal tissue. In addition, 5 pairs of cancer and adjacent normal tissues were collected from the Second Affiliated Hospital of Shantou University Medical College, Shantou, China. This study has been approved by Shantou University medical college Ethic Committee. All methods were performed in accordance with the relevant guidelines and regulations. Informed consent was obtained from all participants and/or their legal guardians.

### Immunohistochemistry

Immunohistochemistry was carried out following an established protocol [31]. Briefly, liver tissue microarray or paraffin tissue sections were dewaxed, rehydrated through graded ethanol and incubated with 3% hydrogen peroxide for 30 min. Antigen retrieval was performed by heating the sections to 95 °C in 0.01 mol/l citrate buffer (pH 6.0) for 15 min. Slides were then washed in PBS for 15 min and treated with 10% normal horse serum for 30 min and incubated with primary antibody at 4 °C overnight. The reaction products were detected with 3-amino-9-ethylcarbozole (AEC) substrate-chromogen kit after incubating with the secondary antibody of Dako REAL EnVision Detection Kit (Dako, Carpinteria, CA) for 30 min and washing in 0.1 M PBS at room temperature. Staining with AEC resulted in red signals. The primary antibody for GLUT2 (Novus Biologicals, NBP2-22218SS, USA), GLUT1 (Abcam, ab115730, USA), were used at a 1:500 dilution and anti-ChREBP (Novus Biologicals, NB400–135, USA) was used at a 1:200 dilution. The antibodies against GLUT2 and ChREBP were rabbit-derived polyclonal and the antibody against GLUT1 was rabbit-derived monoclonal. For negative controls, the primary antibodies were replaced with PBS.

### Stain-Decolorize-Stain (SDS) Method for Liver Microarray

The stain-decolorize-stain method was performed to show whether GLUT2 and ChREBP are expressed in the same hepatocytes/pattern according to an established procedure [32]. Briefly, immunohistochemistry of ChREBP was first performed on the liver microarray. After visualization and taking photos, the slides were decolorized with 80% alcohol for half an hour at room temperature and then heated in a microwave oven for 10 min to remove the bound antibodies. The microarray and slides were then incubated with GLUT2 antibody at 4 °C overnight. After visualization with the AEC kit, photos were taken at the same fields as for the first immunostaining. Appropriate controls were carried out and the specificity of this technique has been reported previously [32].

### Semiquantition of Stainings of ChREBP and Glucose Transporters

Semiquantitative scoring was carried out as described in a previous study [33]. Briefly, tissue microarray sample ‘spots’ were viewed at 400× magnification and an overall score was assigned according to intensity and area of positive immunostaining. Sample scorings are as follows: 0, no red staining at all (negative); 1, pink staining in the minority of tissue (mild); 2, pink staining in the majority of the tissue (middle); 3, red staining in the majority of the tissue (strong) and 4, dark-red staining in all the tissue (very strong) following a well established protocol [33]. To reduce the variation of scoring, all slides, including microarray, were scored by two observers independently (Y. L. and Q. H.).

### Statistical Analysis

The effect of HCC malignancy on protein expression was analyzed with one-way ANOVA. The correlation between 2 protein expression levels were analyzed with Pearson Correlation test. If protein expression levels were normally distributed and had equal variance, Student’s t test was used. If the scores were not normally distributed, we used the Mann-Whitney U test. *p* < 0.05 was considered statistically significant. All figures and statistics were made with Graphpad Prism 7 (CA, USA).

## Results

### Patient Characteristics

A total of 70 liver samples were analyzed, of which 40 were defined as HCC. Mean age of the 40 cancer patients was 49 years and 82% were males. Stages I, II and III accounted for 6%, 19% and 33% of total liver samples, respectively. There were 14 normal liver tissues and 13 cancer adjacent tissues, and they accounted for 20% and 19% of total, respectively (Table 1).

**Table 1:** Patient information

Total samples		70
Average age of cancer patient (mean ± SD)		49 ± 10
	Number	Percentage (%)
Male with cancer	33	82%
Female with cancer	7	18%
Normal tissue	14	20%
Adjacent normal tissue	13	19%
Stage I	4	6%
Stage II	13	19%
Stage III	23	33%
Fatty degeneration	3	4%

### ChREBP Protein Expression Tended to Increase with Malignancy

**Fig. 1:**
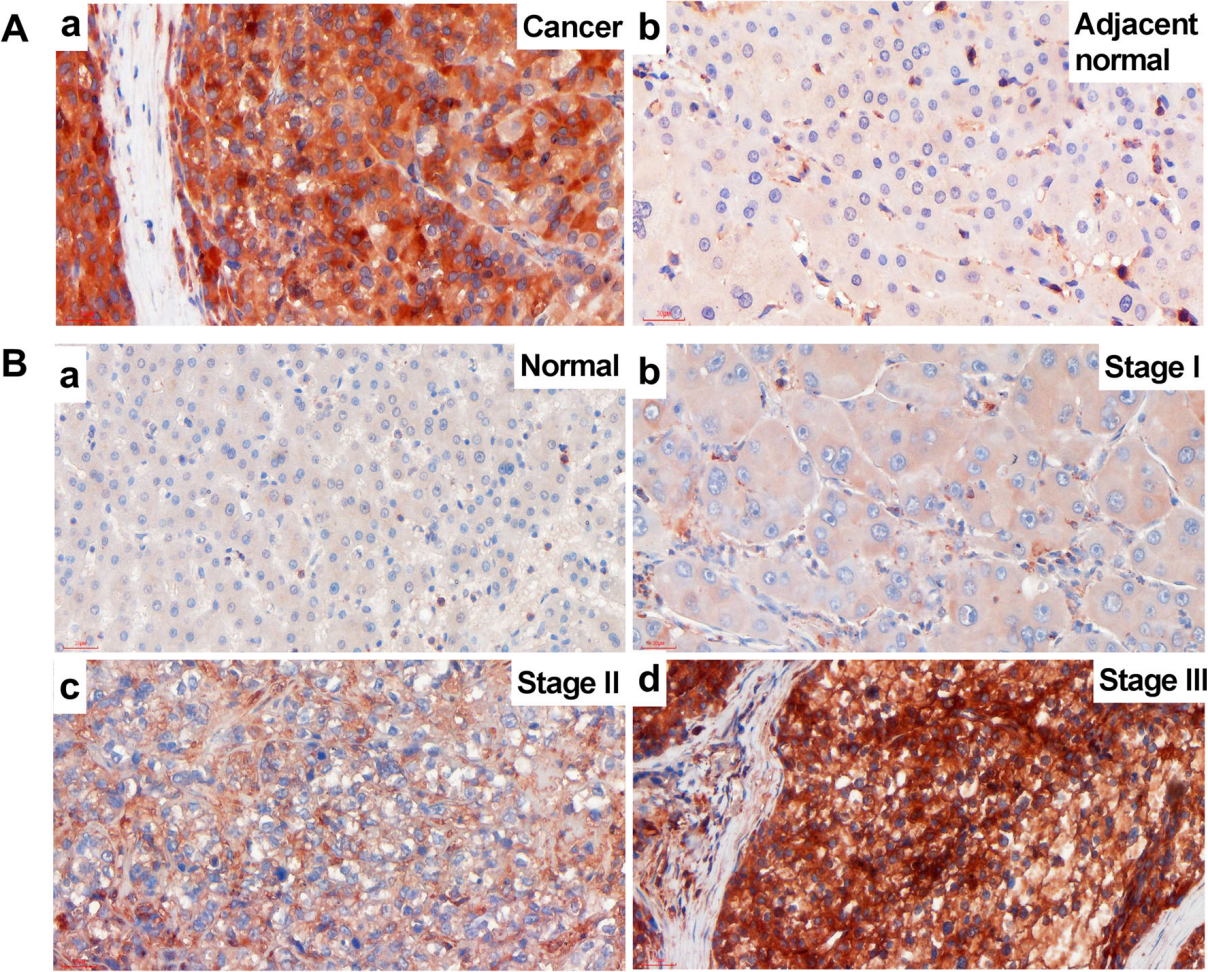
**ChREBP immunohistochemistry in HCC and normal liver tissue. A** The strong ChREBP positive staining in malignant liver (**a**) compared with mild staining in adjacent normal tissue (**b**) in a same patient. Scale bar = 30 μm. **B** ChREBP staining in liver cancer tissues with different malignant progression. a, normal liver tissue; b, stage I liver cancer; c, stage II liver cancer; d, stage III liver cancer. Scale bar = 30 μm

Immunohistochemistry demonstrated that 4 out of 5 pairs of liver sample clearly showed stronger positive intensity of ChREBP protein in malignant tissues than the adjacent normal tissues (Fig. 1a). Only 1 pair of tissue showed similar intensity of ChREBP expression in malignant and adjacent normal tissue. The scatter positive signals in non-hepatocytes were T lymphocytes. All the CD3, T lymphocyte marker, −positive cells showed positive signals for ChREBP (Fig. S1A-B). Most of the CD68, macrophage marker, −positive cells did not show clear ChREBP signals, but a few cells showed mild positive signals (Fig. S1C-D).

ChREBP immunohistochemistry was also performed in tissues of different HCC clinical stages to determine whether there is an association between ChREBP expression and malignancy. The staining results showed a trend of increasing intensity of ChREBP with HCC malignant progression as defined by histopathological diagnosis (Fig. 1b). The staining positivity was quantified with a score defined by staining intensity and size of positive area (Table 2). In normal and HCC adjacent tissues, about 40% of the sections showed no ChREBP positivity and about 37% showed mild ChREBP positivity, but no sections showed strong or very strong stainings. However, in stage II and stage III HCC tissues, about 30% of the sections showed no ChREBP expression, 25% showed mild staining, 9% showed strong expression, and 16% showed very strong positivity (Table 2). The strong and very strong stainings were only seen in highly malignant (Stages II and III) HCC tissues (Fig. 4a and Table 2). However, the result of one-way ANOVA of ChREBP expression and clinical stage found no significant difference (*p* = 0.172). Therefore, there was only a tendency of increased ChREBP expression with malignancy.

**Table 2:** Expression of ChREBP, GLUT2 and GLUT1 in different clinical stages

ChREBP expression in different clinical stages	Negative (0)	Mild (1)	Middle (2)	Strong (3)	Very strong (4)
Normal	36%	36%	28%	0%	0%
Adjacent	46%	39%	15%	0%	0%
I	50%	25%	25%	0%	0%
II	31%	23%	23%	8%	15%
III	30%	26%	17%	9%	18%
GLUT2 expression in different clinical stages					
Normal	7%	14%	36%	21%	22%
Adjacent	15%	15%	39%	31%	0%
I	100%	0%	0%	0%	0%
II	54%	23%	15%	8%	0%
III	61%	26%	9%	4%	0%
GLUT1 expression in different clinical stages					
Normal	64%	36%	0%	0%	0%
Adjacent	69%	23%	8%	0%	0%
I	25%	50%	25%	0%	0%
II	8%	31%	23%	38%	0%
III	9%	26%	35%	26%	4%

### GLUT2 Protein Expression Negatively Correlates to Malignancy

**Fig. 2:**
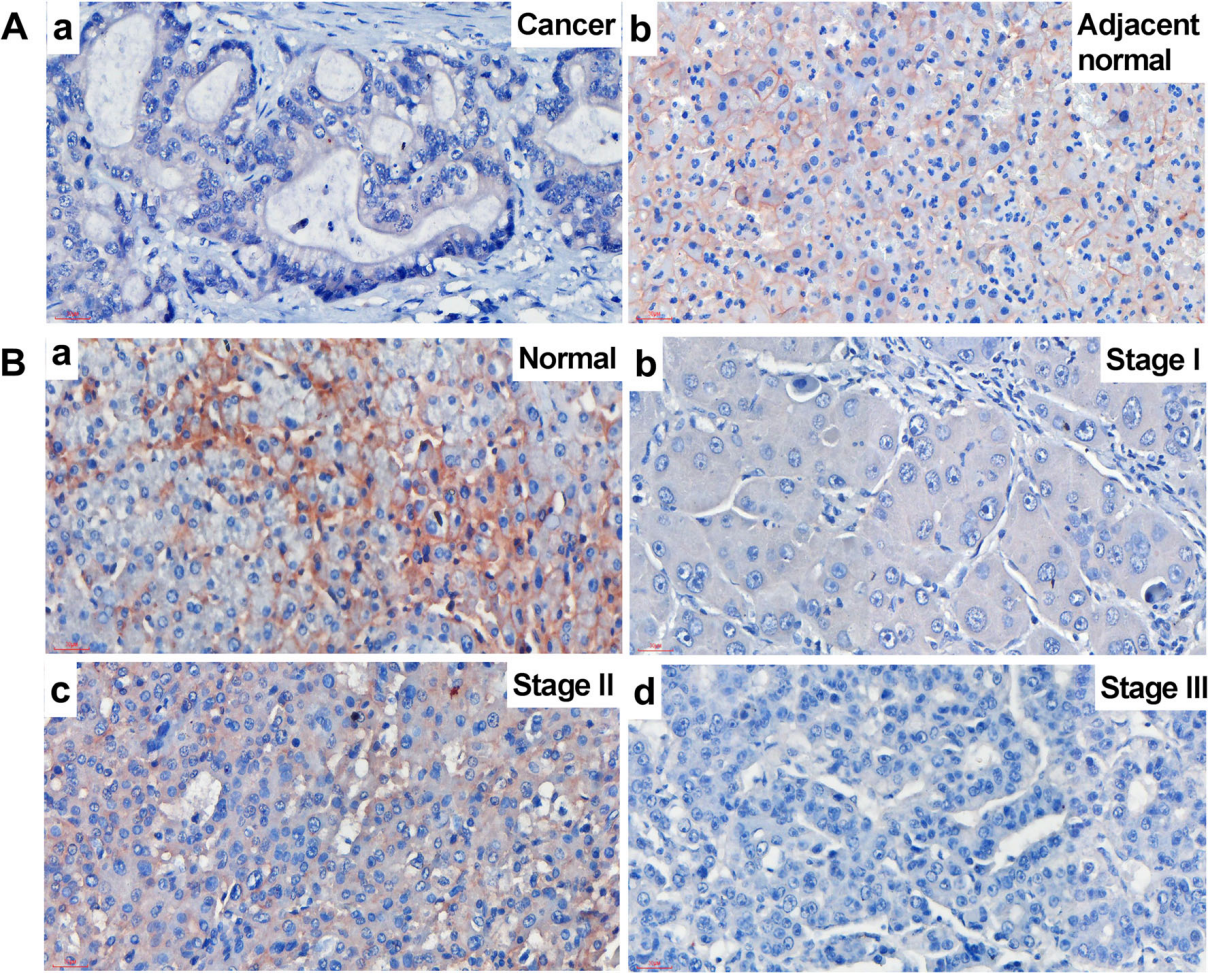
**GLUT2 immunohistochemistry in HCC and normal liver tissue. A** The GLUT2 staining was negative in liver cancer (**a**) but positive on the membrane of the adjacent normal hepatocytes (**b**). **B** GLUT2 staining in liver cancer tissues with different malignant progression. a, normal liver tissue; b, stage I liver cancer; c, stage II liver cancer; d, stage III liver cancer. Scale bar = 30 μm

Since it has been reported that glycolysis is increased in various cancer cells [34, 35] and GLUT2 is the main glucose transporter in liver [18], GLUT2 protein expression was examined (Fig. 2). In HCC, no GLUT2 protein expression was detected (Fig. 2A, a), while GLUT2 was highly expressed in the adjacent normal liver tissue, mainly at the hepatocyte membrane (Fig. 2A, b). In addition, GLUT2 expression was decreased with malignant progression (Fig. 2B and Fig. 4b). In normal hepatocytes, GLUT2 protein was clearly detectable at the hepatocyte membrane (Fig. 2B, a). In stage I liver cancer tissues, no positive signal could be detected (Fig. 2B, b). In stage II, most of the samples showed mild positivity (Fig. 2B, c), but in stage III most tissues showed no signal (Fig. 2B, d). Quantification of stain positivity was carried out based on the positivity scores that were defined by staining intensity and size of positive area (Table 2 and Fig. 4b). It showed that in normal tissue about 36% showed medium GLUT2 positivity, 40% showed strong or very strong staining. Only a few normal liver tissue sections showed negative or mild staining. In stage I HCC, 100% of the sections showed no staining. This may be attributed to limited number of samples in Stage I. In stage II HCC, about 77% of the sections showed negative or mild GLUT2 staining. In stage III HCC, about 90% of the sections showed negative or mild GLUT2 staining (Fig. 4b). The result of one-way ANOVA of GLUT2 protein expression and clinical stage revealed that GLUT2 protein expression negatively correlates to liver malignancy (*P* < 0.001) (Fig. 4b).

### GLUT1 Protein Expression Positively Correlates to Liver Malignancy

**Fig. 3:**
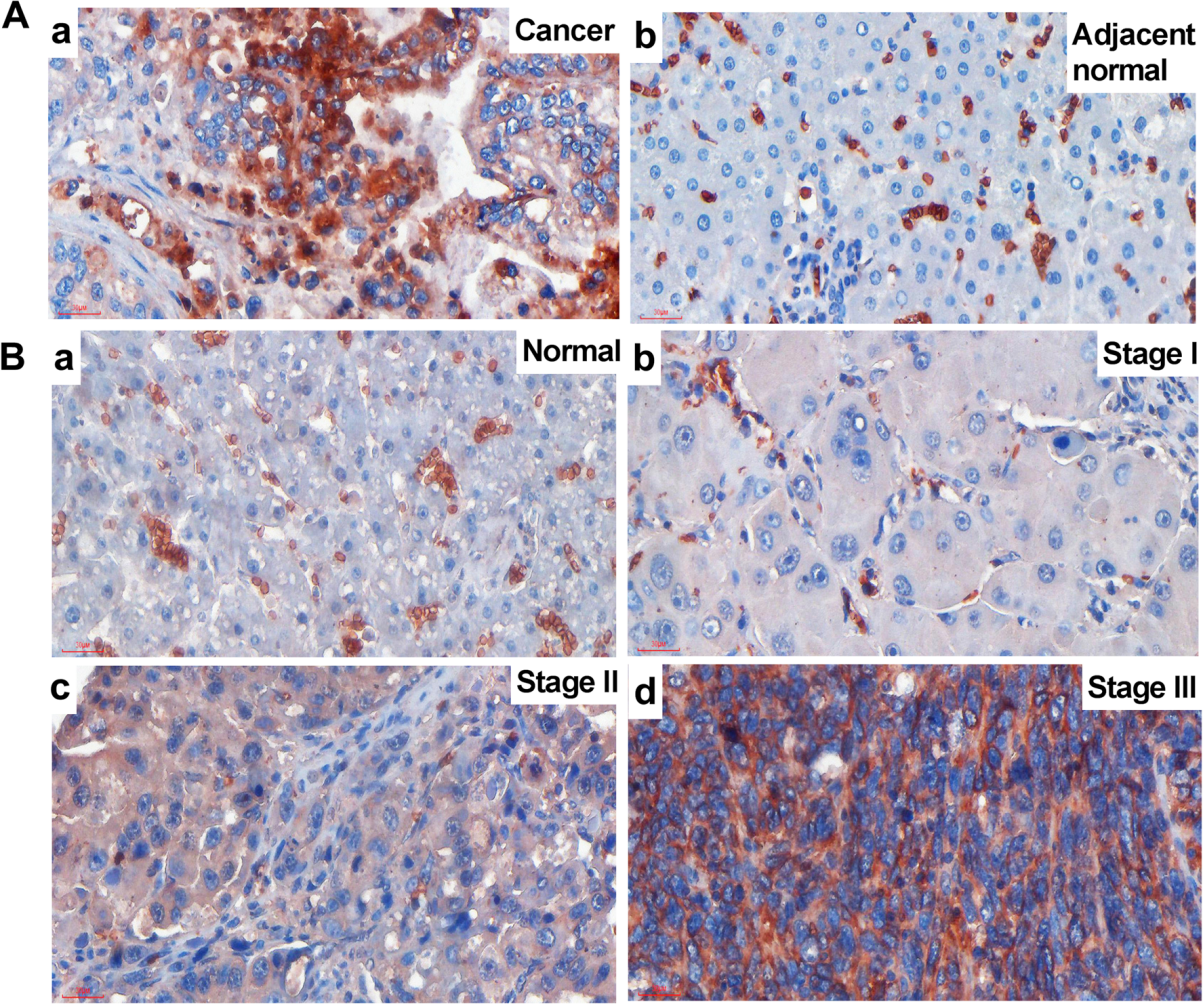
**GLUT1 immunohistochemistry in HCC and normal liver tissue. A** The GLUT1 staining was positive on the membrane and in the cytoplasm in malignant hepatocytes (**a**) but negative in the adjacent normal hepatocytes (**b**). **B** GLUT1 staining in liver cancer tissues with different malignant progression. a, normal liver tissue; b, stage I liver cancer; c, stage II liver cancer; d, stage III liver cancer. Scale bar = 30 μm

**Fig. 4:**
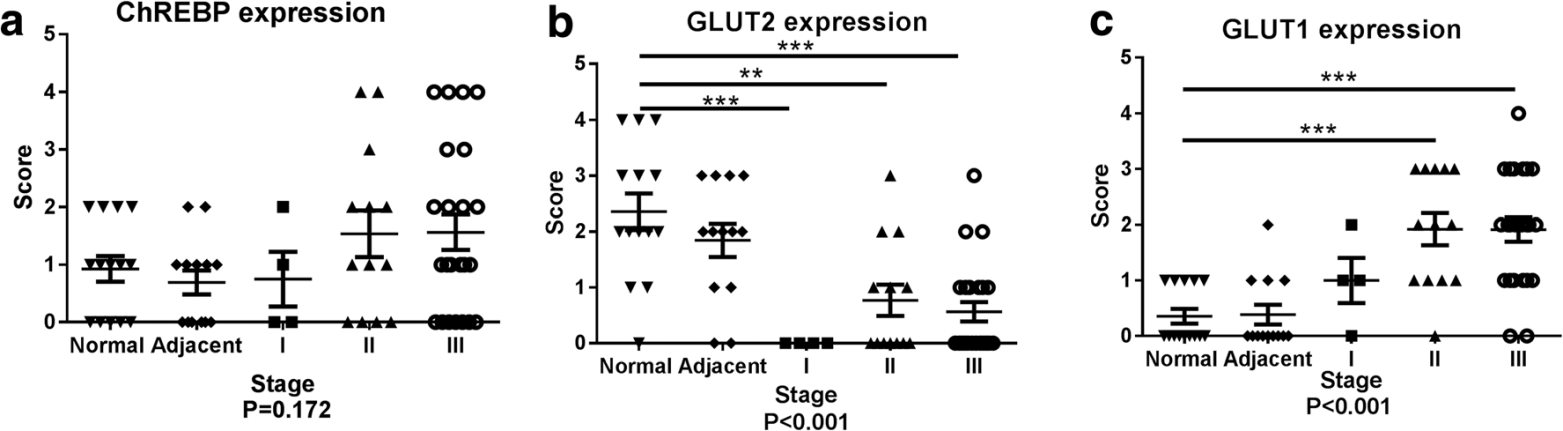
**Histograms of protein expression in different liver tissues.** Histograms of scores of ChREBP (**a**), GLUT2 (**b**), GLUT1(**c**) expression levels in different stages of liver cancer. 0, absent of positive signal; 1, mild staining; 2, middle staining; 3, strong staining; 4, very strong staining. One-way ANOVA was used to analyze the correlation between protein expression and liver malignancy. Student-T test or Mann-Whitney U test was used to analyze the protein expression differences between 2 different clinical stages. **, *P* < 0.01; ***, *P* < 0.001

Since it has been reported that GLUT1 expression might increase to contribute to increased glycolysis in some cancers [36], we examined GLUT1 expressions on the liver tissues. We found that GLUT1 expression in HCC was significantly higher than that of the adjacent normal tissue (Fig. 3a). The non-hepatocyte positive cells were red blood cells because of the lacking of nuclei (Fig. S2). Besides, GLUT1 expression was increased with malignant progression (Fig. 3b and Fig. 4c). In normal hepatocytes, no GLUT1 positive signal could be detected (Fig. 3B, a). In stage I liver cancer tissues, only weak positive signals were detected (Fig. 3B, b). In stage II, most of the samples showed mild positivity (Fig. 3B, c), and in stage III, most hepatocytes showed strong positive signals on cell membrane and in cytoplasm (Fig. 3B, d). Quantification of the staining intensity (Table 2) showed that in normal tissues, only 36% showed mild positivity and majority (64%) of the sections showed no GLUT1 staining. In HCC adjacent tissues, similar expression pattern was observed to that in the normal tissues. In stage I HCC, 25% of the sections showed negative staining, while mild positivity represented 50% and medium positivity 25%. No strong or very strong positivity was observed in stage I HCC. In stage II HCC, however, 8% of the sections showed GLUT1 negative expression, 31% showed mild positivity, 23% showed medium positivity and 38% showed strong positivity. In stage III HCC, 9% of the sections showed GLUT1 negative expression, 26% showed mild positivity, 35% showed medium positivity and 30% showed strong and very strong positivity (26% strong, 4% very strong) (Fig. 4c). The result of one-way ANOVA of GLUT1 expression and clinical stage showed that GLUT1 protein expression was increased significantly (*P* < 0.001) following malignant progression of HCC (Fig.4c).

### Correlations between Different Protein Expression Levels

To study the relationships among expressions of ChREBP, GLUT1 and GLUT2, the Pearson Correlation test was performed between any two different proteins. Although ChREBP had the trend to increase and GLUT2 expression decreased with HCC malignant progression, there was no correlation between these 2 proteins (r = −0.031, *p* = 0.796, *n* = 70). However, GLUT1 expression was significantly positively correlated with ChREBP expression, and both proteins increased with HCC malignancy (r = 0.481, *p* < 0.0001, n = 70). GLUT1 expression was inversely associated with GLUT2 expression (r = −0.320, *p* = 0.007, n = 70).

### GLUT1 but Not GLUT2 Protein Co-Expressed with ChREBP in HCC

**Fig. 5:**
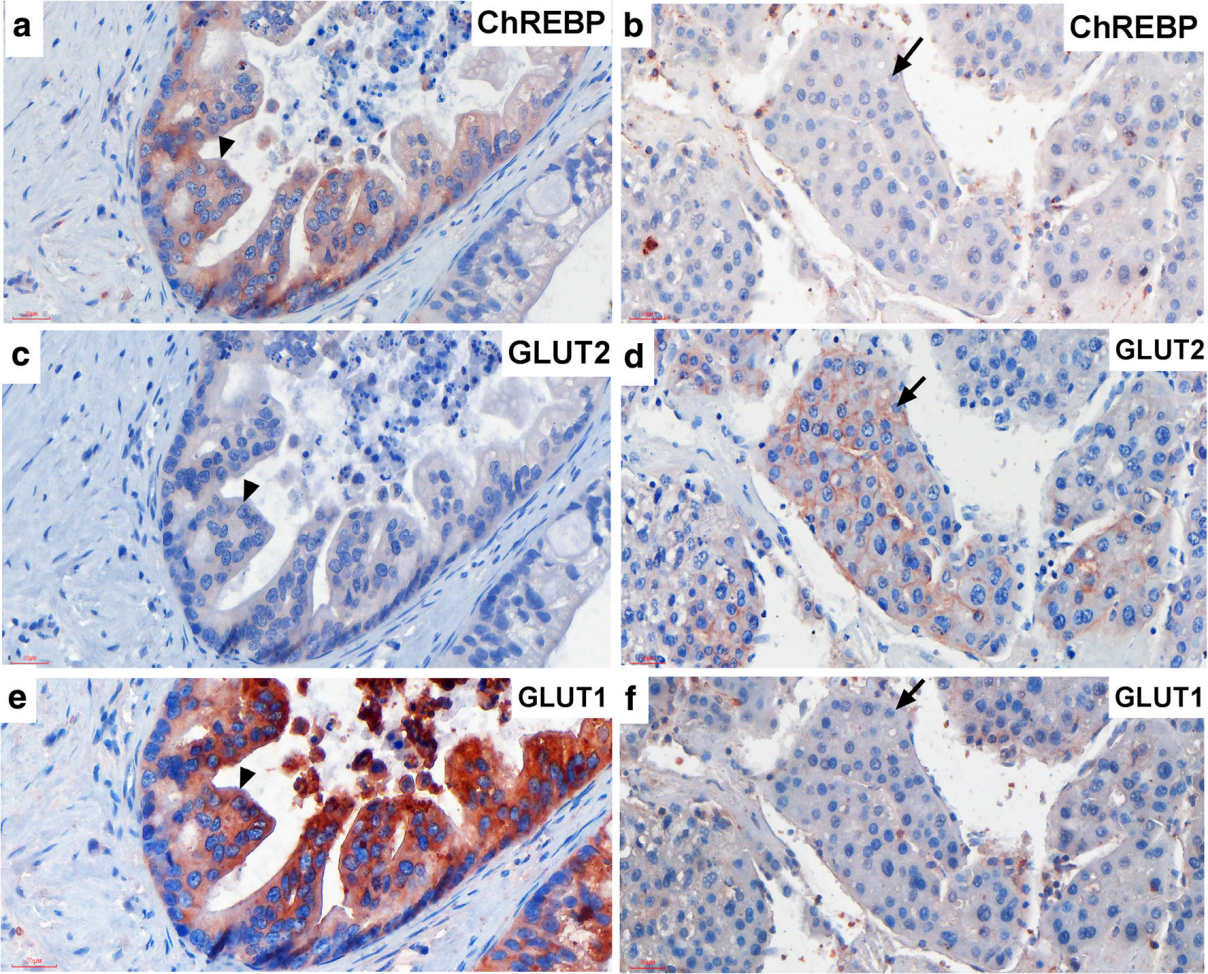
**GLUT1 but not GLUT2 protein co-expressed in ChREBP-positive hepatocytes in HCC**. Immunohistochemistry of ChREBP-positive malignant hepatocytes (**a**) showing GLUT2 negative (**c**) but GLUT1 positive (**e**) staining. Arrow heads indicating the same cell. Immunohistochemistry of ChREBP-negative malignant hepatocytes (**b**) showing GLUT2 positive on the membrane (**d**) but GLUT1 negative staining (**f**). Arrow indicating the same cell. Scale bar = 30 μm

To further investigate ChREBP, GLUT2 and GLUT1 protein expression patterns, stain-decolorize-stain method was performed. The results showed that GLUT1 but not GLUT2 positive staining could be detected in ChREBP-positive hepatocytes (Fig. 5a c and e). However strong GLUT2 but not GLUT1 positive staining was detected in ChREBP-negative hepatocytes (Fig. 5b, d and f). All slides showed that ChREBP and GLUT1 but not GLUT2 co-expressed in the same cells.

## Discussion

It has been proposed that the glucose-sensitive transcription factor ChREBP plays a role in cancer pathology. It has been reported that suppression of ChREBP mRNA inhibited cell proliferation in vitro and reduced tumor growth in vivo [29]. ChREBP protein expression was positively correlated to breast cancer malignancy, i.e., more malignant cancers express more ChREBP protein [30]. However, in hepatocellular carcinoma, the relationship between ChREBP expression and cancer malignancy has not been established. In this study, we found that ChREBP protein expression tended to increase with cancer progression although, with the limited number of samples, could not reach statistical significance in compassion to normal liver tissue. In normal liver tissue, there was no or weak ChREBP protein expression, but in liver cancers the expression was abundant. In stage III HCC patients, 32% of the samples showed strong staining of ChREBP. The increased ChREBP expression may serve to support increased glycolysis and lipogenesis in cancerous hepatocytes. Although ChREBP protein levels can be used as a novel diagnostic and prognostic marker in breast cancer [30], variation in HCC appears to be too large for this purpose. Nevertheless, ChREBP may contribute to diagnosis of cancer grading in combination with other markers.

Most cancer cells have increased aerobic glycolysis under both anaerobic and aerobic conditions [37]. This increased glycolysis, accompanied by accelerated glucose uptake, is known as the Warburg effect, after the German biochemist Otto Warburg who first described the phenomenon in 1920s [38]. Increased glucose uptake is contributed by transmembrane transport mediated by specific glucose transporters [39, 40] and increased activity of hexokinases [41, 42]. Many studies have demonstrated that the expression of glucose transporters, especially GLUT1, was increased in a variety of malignancies and GLUT1 overexpression was associated with invasiveness and poor overall survival of various malignant tumors [43–47]. GLUT2 mRNA expression was inversely associated with overall survival in HCC [18] and invasiveness in insulinomas [20]. Thus our data confirm that GLUT2 can be potentially used for HCC diagnosis and prognostic prediction [18]. It has been reported that GLUT2 is a target of ChREBP [22], but its expression had an opposite pattern compared to ChREBP in our study. The possible reason could be that GLUT2 is not only regulated by ChREBP but also that other regulators could have stronger influences on GLUT2 expression. This notion, however, requires further investigation.

It has been reported that the increased glucose transport was due, at least in part, to increased expression of GLUT1 in cancers compared to normal tissues [36] and that GLUT2 was replaced by GLUT1 in human hepatocellular carcinoma cell line HepG2 [48]. These results suggest that the increased expression of GLUT1 gene may closely relate to cellular transformation. In our study, we found that GLUT1 was significantly increased in HCC compared to adjacent normal hepatocytes and its expression was positively associated with HCC progression. Therefore, GLUT1 might serve as a diagnostic marker for HCC. Moreover, we found that ChREBP-expressing hepatocytes do not express GLUT2 protein but express GLUT1, and that ChREBP-negative hepatocytes express GLUT2 but not GLUT1. This indicates that ChREBP and GLUT1 have a similar expression profile. Upon statistical analysis, we found that ChREBP and GLUT1 protein levels were significantly positively correlated to each other and GLUT1 and GLUT2 levels were significantly reversely associated. Different glucose transporters have different binding affinities for glucose. GLUT1/HKIV has high affinity for glucose [46] but GLUT2/GCK has relatively low affinity for glucose, mannose and galactose [47]. Therefore, a transition from GLUT2/GCK to GLUT1/HKIV mediated glucose metabolism could contribute to cancer differentiation because of losing sensitivity of the liver to circulating glucose levels.

Taken together, this is to our best knowledge the first to report of ChREBP expression in HCC and its relationship with glucose transporters GLUT1 and GLUT2. Analysis of combined profile of ChREBP, GLUT1 and GLUT2 expression should be helpful for HCC diagnosis and shed light on improvement of HCC treatment and patient survival.

## Electronic supplementary material

ESM 1 (DOCX 4898 kb)
