# Supplementary material for: Expressions of Carbohydrate Response Element Binding Protein and Glucose Transporters in Liver Cancer and Clinical Significance
Source: Pathol Oncol Res. 2019 Aug 12;26(2):1331–40. doi: 10.1007/s12253-019-00708-y (PMC7242283; doi:10.1007/s12253-019-00708-y)
Supplement: Supplementary file 1 — (DOCX 4898 kb) [file 12253_2019_708_MOESM1_ESM.docx]

**Figure S1**


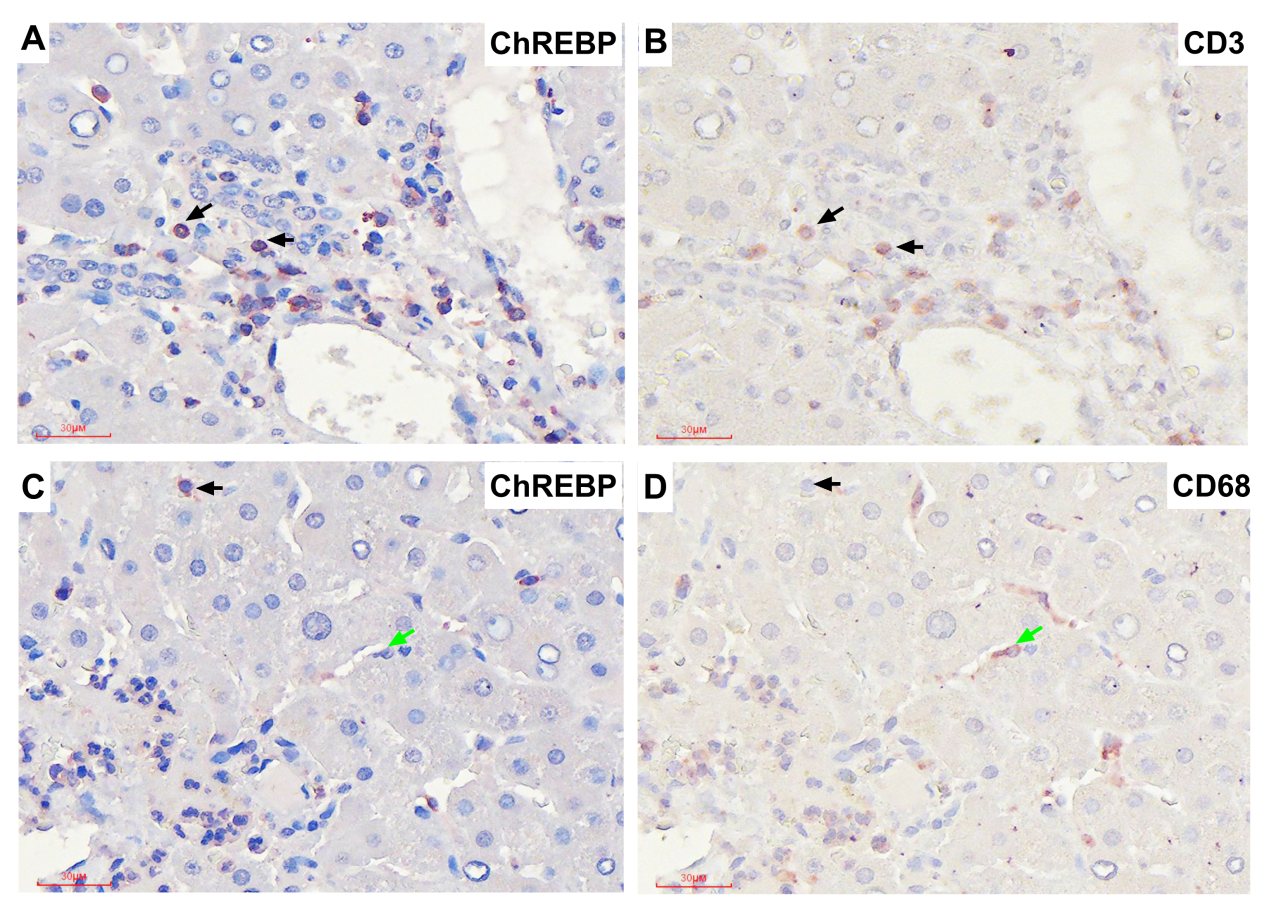


**Figure S1. T lymphocytes but not Kuppfer cells express ChREBP in normal liver tissue**

Immunohistochemistry of CD3-positive（B）T lymphocytes showing ChREBP positive (A) but CD68-positive Kupffer cells (D) showing ChREBP negative (C) staining. Arrow heads indicating the same cell. Scale bar=30 μm.

**Figure S2**


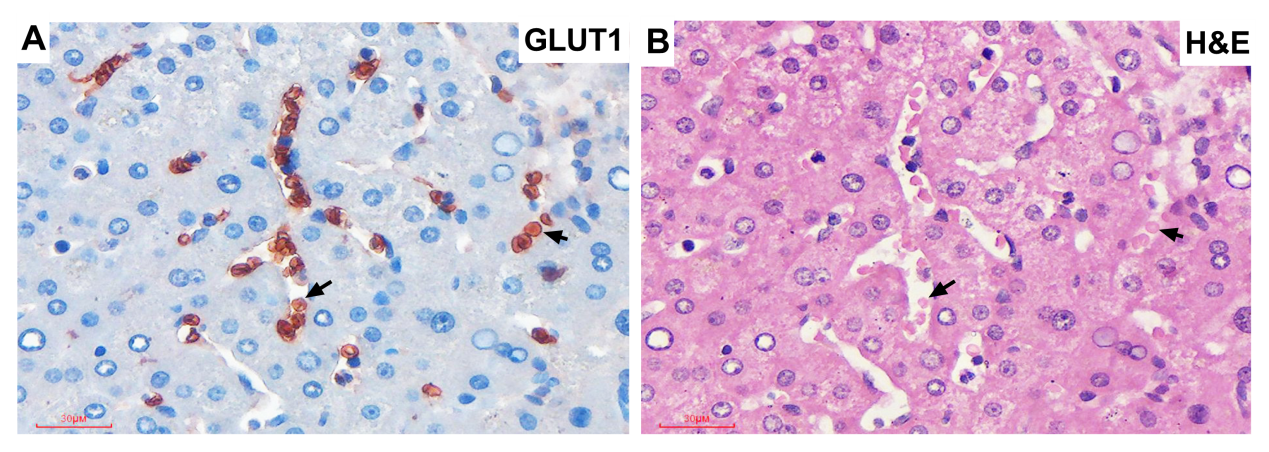


**Figure S2. Red blood cells express GLUT1 in normal liver tissue**

A, Immunohistochemistry of GLUT1 in normal liver tissue. B, H&E staining of normal liver tissue. Arrow heads indicating the same cell. Scale bar=30 μm.

**Materials and Methods**

**Immunohistochemistry**

Immunohistochemistry was carried out following an established protocol as described in the main manuscript. The primary antibodies for ChREBP (Novus Biologicals, NB400-135, USA), CD3 (ZSGB-Bio, ZM-0417, China), and CD68 (ZSGB-Bio, ZM-0060, China) were used at a 1:200 dilution and GLUT1 (Abcam, ab115730, USA ) was used at a 1:500 dilution. For negative controls, the primary antibodies were replaced with PBS.

**H&E staining**

H&E staining was conducted to identify GLUT1-positive cell type in hepatic sinusoid according to routine procedures. Brieﬂy, paraffin-embedded tissue sections were deparaffinized in xylene and rehydrated in a graded series of alcohol. Then sections were stained with hematoxylin for 5 minutes followed by 5 dips in 1% acid ethanol and then rinsed in water. Sections were subsequently stained with eosin for 3 minutes and followed by dehydration with graded alcohol and clearing in xylene.

**Stain-decolorize-stain (SDS) method**
The stain-decolorize-stain method was performed to show which type of cells express ChREBP in liver tissue according to an established procedure as described in the main manuscript. Briefly, immunohistochemistry of ChREBP was first performed on the liver tissue slides. After visualization and taking photos, the slides were decolorized with 80% alcohol for half an hour at room temperature and then heated in a microwave oven for 10 min to remove the bound antibodies. The slides were then incubated with CD20 and CD68 antibodies at 4 ˚C overnight. After visualization with the AEC kit, photos were taken at the same fields as for the first immunostaining.
